# Harveian Oration 2024: From bench to bedside and beyond – new horizons for translational ageing research

**DOI:** 10.1016/j.clinme.2025.100334

**Published:** 2025-06-03

**Authors:** Avan Aihie Sayer

**Affiliations:** a AGE Research Group, Translational and Clinical Research Institute, Faculty of Medical Sciences, Newcastle University, Newcastle upon Tyne, UK; b NIHR Newcastle Biomedical Research Centre, Newcastle upon Tyne Hospitals NHS Foundation Trust, Cumbria Northumberland Tyne and Wear NHS Foundation Trust and Newcastle University, Newcastle upon Tyne, UK; c Multiple Long-Term Conditions Cross-NIHR Collaboration, Newcastle University, Newcastle upon Tyne, UK

**Keywords:** Ageing, Sarcopenia, Translational ageing research, Geriatric medicine, Older people

## Introduction

It is a great honour to deliver the Royal College of Physicians’ Harveian Oration and a wonderful opportunity to share progress in the new field of translational ageing research. I like the phrase ‘standing on the shoulders of giants’, by which I mean acknowledging and using the wisdom of those who have gone before. This means not only giants in the field of ageing, but also Harveian orators dating back to 1656 when the tradition was established by William Harvey. Here I focus on three aspects of ageing. Firstly ‘the bench’, shorthand for the biology of ageing; secondly ‘the bedside’, shorthand for translating biology findings into studies in people, and thirdly ‘beyond the bench and bedside’, referring to a lifecourse approach to ageing. I finish with where I see opportunities ahead.

My clinical career has been in geriatric medicine, specialising in the healthcare of older people and early on, it became apparent that the process of becoming older could vary widely. To understand this, I found the concept of biological as opposed to chronological age helpful. Biological ageing can be defined as deteriorative changes with time during post-maturational life that underlie an increasing vulnerability to challenges, thereby decreasing the ability of the organism to survive.^1^ And although this definition is nearly 30 years old now, it accords well with how ageing is described in the 2021 House of Lords Inquiry on Ageing.^2^

There is often an instinctive dislike and fear of ageing, which is unsurprising given that it heralds the later stages of life. However it can also lead to unhelpful ageism that pervades much of society, and awareness is important as a first step towards addressing it.^3^ Portraits of William Harvey illustrate human ageing beautifully (Fig. 1), but physical changes are only part of the story. He was born in 1578 and lived to the age of 79. He published his major work on the circulation at the age of 50 and his last work at the age of 73 before establishing the annual Harveian Oration in 1656 a year before he died. For him, ageing was clearly associated with immense academic productivity (Fig. 1).^4^

**Fig. 1(a):**
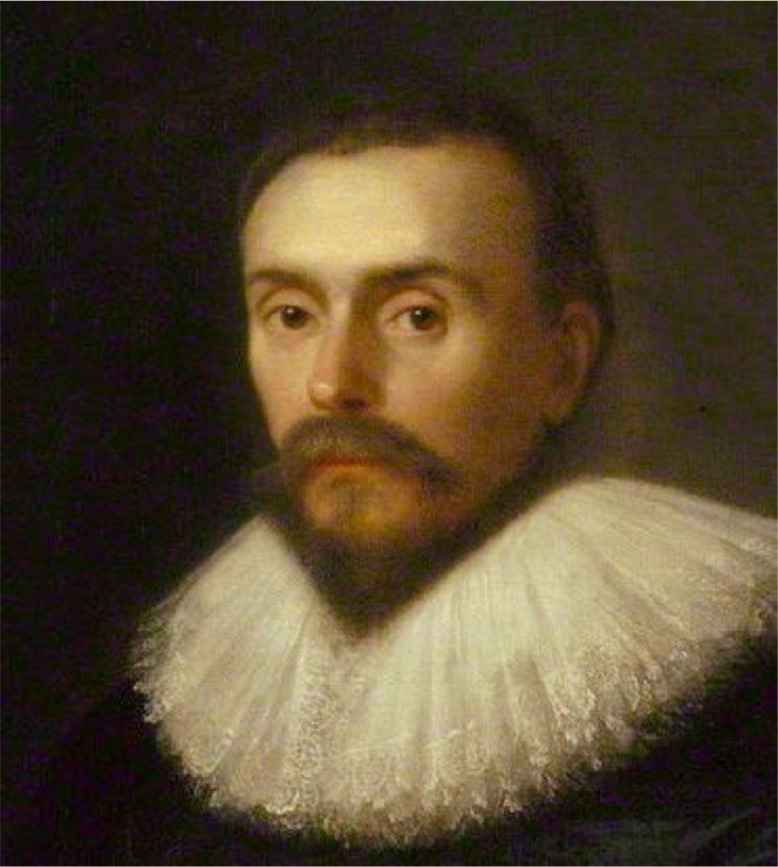
Illustrating biological ageing: portraits of William Harvey at different stages of life. (a) William Harvey circa 1627 attributed to Daniël Mijtens. Reproduced with permission from the National Portrait Gallery London.

**Fig. 1(b):**
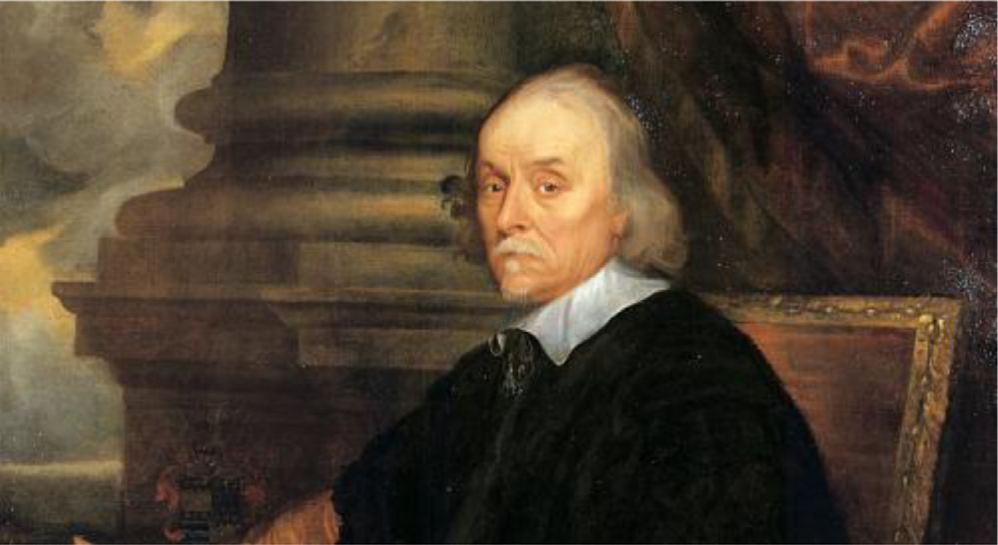
William Harvey circa 1655–1660. Unknown artist, formerly attributed to Cornelius Johnson. Reproduced with permission from the Royal College of Physicians, London.

There are three broad categories of ageing theory: genetic, random event and evolutionary. Evolutionary theories are useful for understanding why ageing occurs. For example, the Disposable Soma Theory proposes that ageing has evolved at a species level because of necessary trade-off in allocation of finite resource between essential processes of reproduction and repair of the soma (body). Optimum investment depends on complexity of the organism and level of environmental danger. In simpler organisms, ageing may be extremely slow, if it occurs at all. In complex organisms, investment of resource required to maintain the body in a perfect state would be so great as to jeopardise reproduction. Reproduction is prioritised and ageing is therefore the result of imperfect maintenance of the body. This understanding is helpful because it predicts that there are likely to be a range of genetic and environmental contributors to ageing.^5^

Ageing matters because there is global population ageing and the impact on the UK is highlighted in the 2023 chief medical officer annual report *Health in an Ageing Society*.^6^ It also describes marked inequality in life expectancy and healthy life expectancy at birth for women from the most and least deprived areas of England. Healthy life expectancy for the most deprived was 51.9 years compared with 70.7 years for the least deprived. However, the report also identified hope in this, because the difference implies that human biological ageing is tractable. Physical activity is beneficial for general health, including ageing, and recent UK guidelines go so far as to say, ‘if physical activity were a drug, we would refer to it as a miracle cure’.^7^ However, not everyone can be active, so alternative and adjunctive approaches are needed.

## The bench: advances in the biology of ageing

Major advances in the biology of ageing include the evolving concept of a hallmark of ageing. This is a biological process that exhibits three characteristics: it changes with time at a molecular or cellular level and accompanies ageing, it causes ageing to accelerate when experimentally increased, and it causes ageing to decelerate, halt or reverse when experimentally reduced. Hallmarks of ageing were the focus of a 2013 landmark paper where nine key hallmarks, including cellular senescence, mitochondrial dysfunction and deregulated nutrient sensing, were identified.^8^ Ten years later, an additional three hallmarks were identified.^9^ Importantly, there are potential interventions to address each of the hallmarks and the geroscience hypothesis proposes that the current medical model of targeting one disease at a time will in time be replaced by a geroscience approach of targeting one ageing hallmark at a time, with the potential to address multiple age-related diseases at once.^10^

Cellular senescence is the focus of much attention. The Hayflick limit is the number of times that a normal somatic, differentiated human cell population will divide before cell division stops and cells become senescent. Historically, senescence was thought to only occur in replicating cells; however, it is now recognised that it can occur in response to stressors such as DNA damage and mitochondrial dysfunction. Therefore, post-mitotic tissues such as skeletal muscle can be affected. When senescent cells accumulate, they not only affect regeneration but also the structure and function of tissue. They also secrete extracellular modulators such as cytokines and growth factors, called the senescence-associated secretory phenotype (SASP), which can affect neighbouring cells and have systemic effects. Cellular senescence may therefore be implicated in age-related diseases such as sarcopenia.^11^

**Fig. 2:**
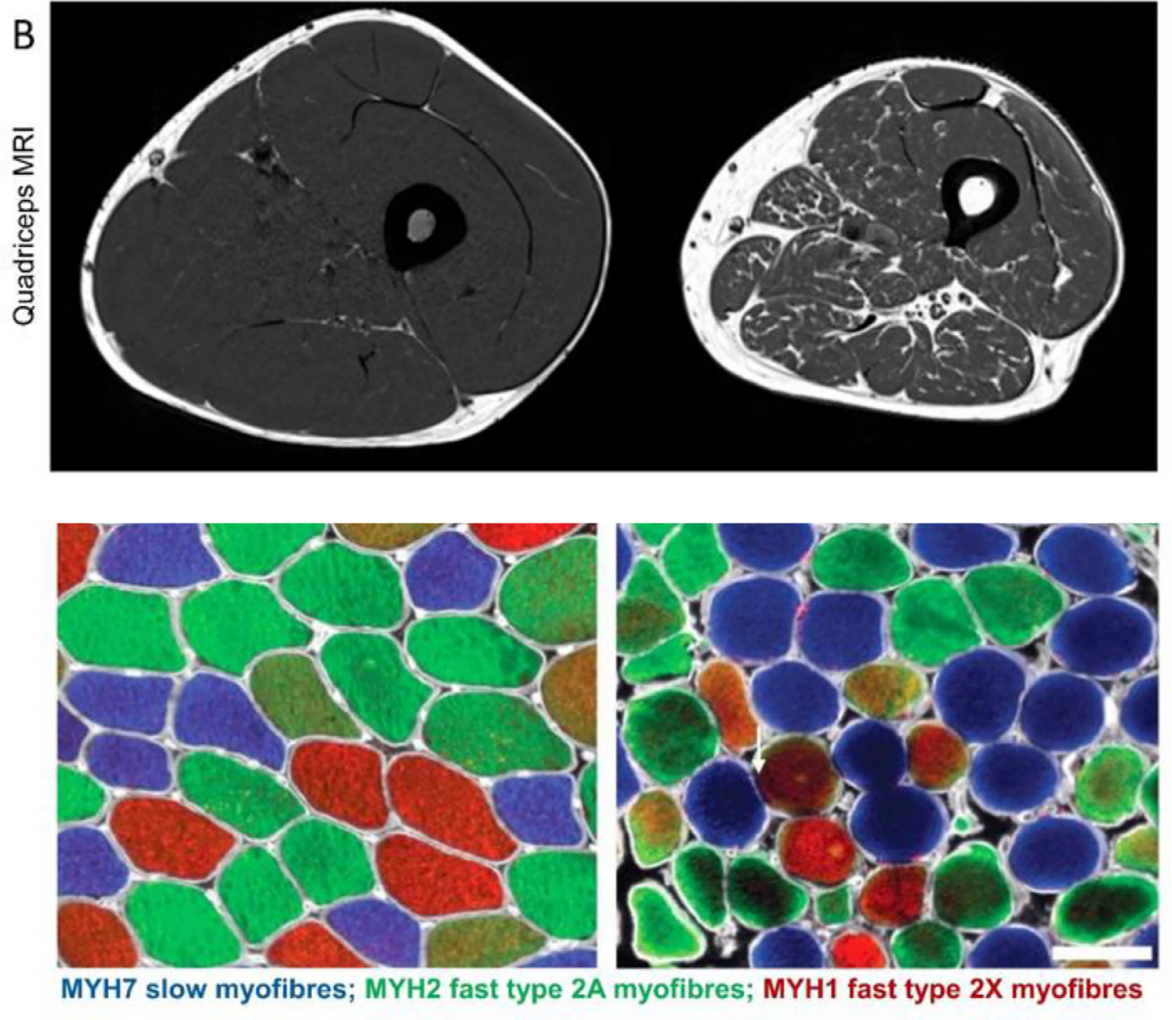
Comparing skeletal muscle at organ and cellular level in a young active individual on the left and an older inactive individual on the right. Used with permission from Granic A *et al*. Hallmarks of ageing in human skeletal muscle and implications for understanding the pathophysiology of sarcopenia in women and men. *Clin Sci* 2023;137:1721–51.

Sarcopenia is the loss of skeletal muscle strength and mass that is commonly seen with advancing age (Fig. 2). It is a relatively newly recognised condition, so there are differing approaches to diagnostic criteria. However, even taking a conservative approach it is common, with a prevalence of 5–10 % in the general population and important because of associations with mobility impairment, morbidity and mortality.^12^ The gold standard treatment for sarcopenia is resistance exercise and ensuring an adequate diet, but there remain no licensed drugs to date.^13^ Better understanding of underlying molecular and cellular processes in skeletal muscle is therefore needed to inform the development of novel interventions.

**Fig. 3:**
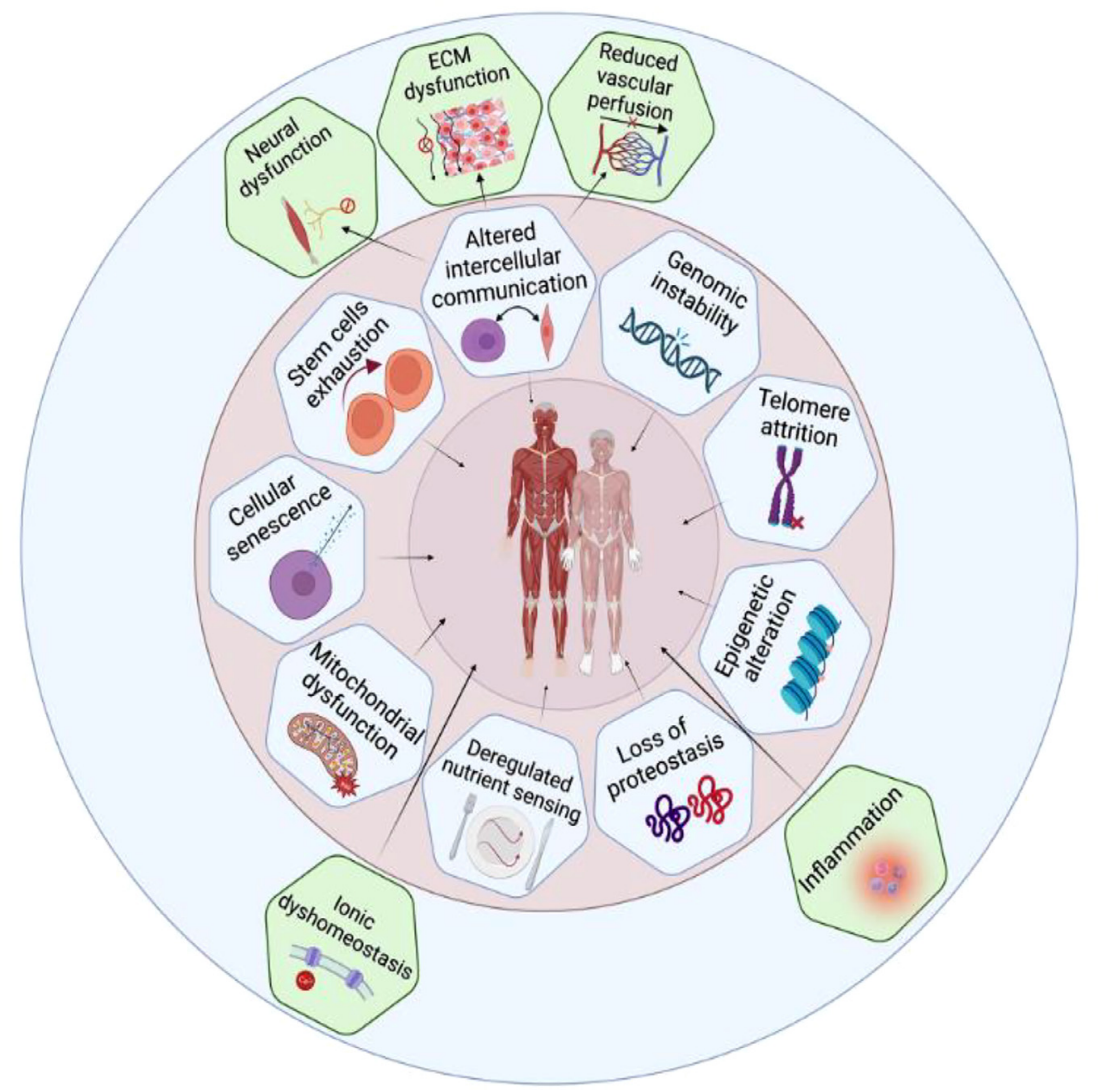
Hallmarks of ageing in human skeletal muscle. Used with permission from Granic A *et al*. Hallmarks of ageing in human skeletal muscle and implications for understanding the pathophysiology of sarcopenia in women and men. *Clin Sci* 2023;137:1721–51.

The evidence for hallmarks of ageing in human skeletal muscle is summarised in a recent review.^14^ In addition to a focus on the nine classic hallmarks of ageing, five new hallmarks of ageing in human skeletal muscle are proposed, namely neuronal dysfunction, extracellular matrix dysfunction, reduced vascular perfusion, inflammation and ionic dyshomeostasis (Fig. 3). Evidence for the role of cellular senescence in skeletal muscle ageing is emerging. A study of young versus old mice demonstrated that older mice had higher p16 and p21 markers of senescence than young mice across muscles. There was also response to a senolytic intervention called DQ, with improved molecular and morphological changes as well as increased grip strength. There were some supportive findings from a small human study.^15^ So, looking ahead, there is potential to scale up the study of human biological ageing to inform the development of interventions.

## The bedside: essentials for translational ageing research

Translational ageing research is relatively unchartered waters for the ageing field, but we have learnt a lot setting up the AGE Research Group in Newcastle and this learning distils into six essentials. **Essential one** is interdisciplinary team science including not only academic geriatricians, but other medical specialties such as neurophysiology and other clinical disciplines such as physiotherapy. The group also comprises a range of scientific disciplines together with skilled research managers, and there has been a transformative effect of having an expert in patient and public involvement. An interdisciplinary team science approach is key to addressing the complexity of ageing, but it has proved no light undertaking to acquire the skills and resource needed to work together effectively.

**Essential two** is research infrastructure to deliver translational ageing research and in the north east, we are fortunate to be well served with National Institute for Health and Care Research (NIHR) infrastructure tailored to a different part of the translational pathway. This includes one of 20 NIHR Biomedical Research Centres across England and the only one to focus on ageing and multiple long-term conditions. **Essential three** is delivering bold studies, working with patients and the public to address gaps where others may not yet see value or only see risk because the approach has not been tried before. An example is MASS Lifecourse, which has built a deep phenotyped cohort for advances in the prevention, diagnosis and treatment of muscle ageing across the lifecourse.^16^ It involves participants aged 18–85 years and includes thigh muscle biopsies, which many thought would be too invasive to be done. Fortunately, the intrepid and altruistic people of the north east proved them wrong and were keen to be involved in pioneering research. Follow-up of the participants is now underway.

**Essential four** is collaboration with academic excellence – particularly important for a relatively new field. We have benefited from national and international collaborations, for example with the Mayo Clinic, to carry out a pilot feasibility study to investigate cellular senescence in human skeletal muscle from initial MASS Lifecourse participants.^17^ **Essential five** is collaboration with industry excellence and an example is the collaboration we have developed with Regeneron Pharmaceuticals looking at molecular and morphometric profiling of human skeletal muscle from MASS Lifecourse.

**Fig. 4:**
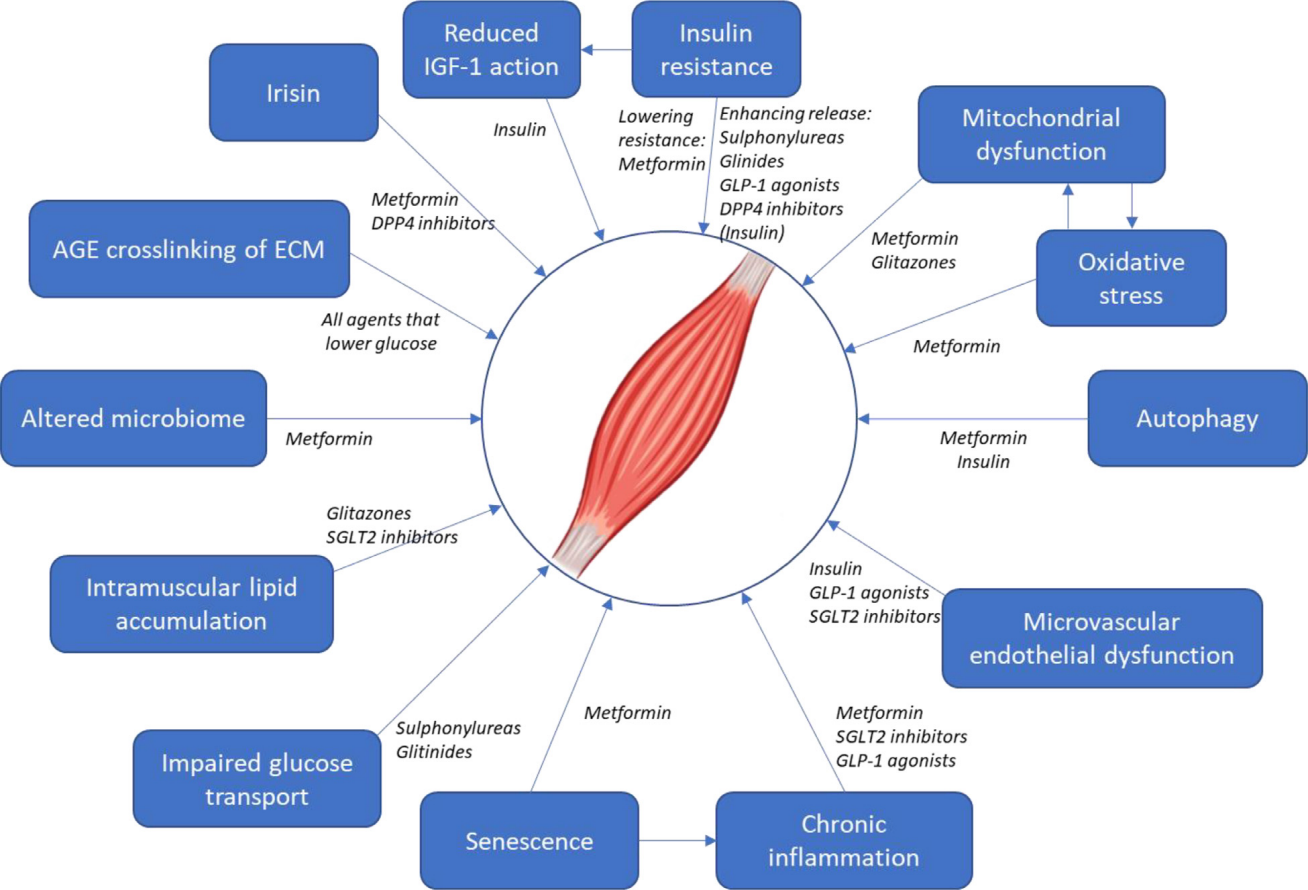
Mechanisms potentially contributing to sarcopenia that may be amenable to modification with drugs used to treat diabetes. Used with permission from Witham MD, et al. Repurposing drugs for diabetes mellitus as potential pharmacological treatments for sarcopenia—a narrative review. Drugs Ageing 2023;40(8):703–719.

**Essential six** is expertise in developing interventions for older people. The gold standard for sarcopenia is resistance exercise, but better understanding is needed of nutritional interventions, new drugs and the potential to repurpose old ones. The diabetes medication metformin has potential. It has multiple different actions not only on insulin resistance but also on mitochondrial function, inflammation and senescence and therefore might be an effective anti-ageing drug (Fig. 4).^18^ This led to a phase II randomised controlled trial called MET-PREVENT to test whether metformin as a candidate geroprotector could improve muscle strength and walking distance in older people living with sarcopenia and frailty. Importantly, this study embedded patient and public involvement from the start, recruited with broad inclusion criteria and few exclusion criteria, and tailored delivery with telephone pre-screening, home visits for screening and the potential for everything to be delivered in participants’ homes.

The findings were striking. Firstly, it proved possible to recruit a frail group of people, with an average age of 80 years and over half using a walking aid, who would not normally be invited to take part in a clinical trial and did this despite the COVID-19 interruption. Secondly, the completion rate was 97 %, much higher than anticipated. Thirdly, metformin did not improve muscle strength, physical performance, quality of life or activities of daily living and there were high rates of adverse events.^19^ Key learning was that the methodological approach, if not the drug, was worth taking forward and this study paves the way for a UK-first and potentially UK-wide ageing platform trial for sarcopenia.

## Beyond the bench and bedside: a lifecourse approach

Ageing is not just about later life; a lifecourse approach to ageing proposes that each stage of life, right from the beginning, has an impact. These ideas first emerged in the epidemiological world from David Barker’s pioneering fetal and infant origins of adult disease hypothesis^20^ that led me to do a PhD investigating whether rates of ageing might be determined *in utero*. This showed for the first time that lower weight in early life was associated with increased signs of biological ageing 60 or 70 years later, including reduced grip strength.^21^ Subsequently, the fetal and infant origins ideas were expanded by others into a lifecourse approach to health and ageing.^22^

**Fig. 5:**
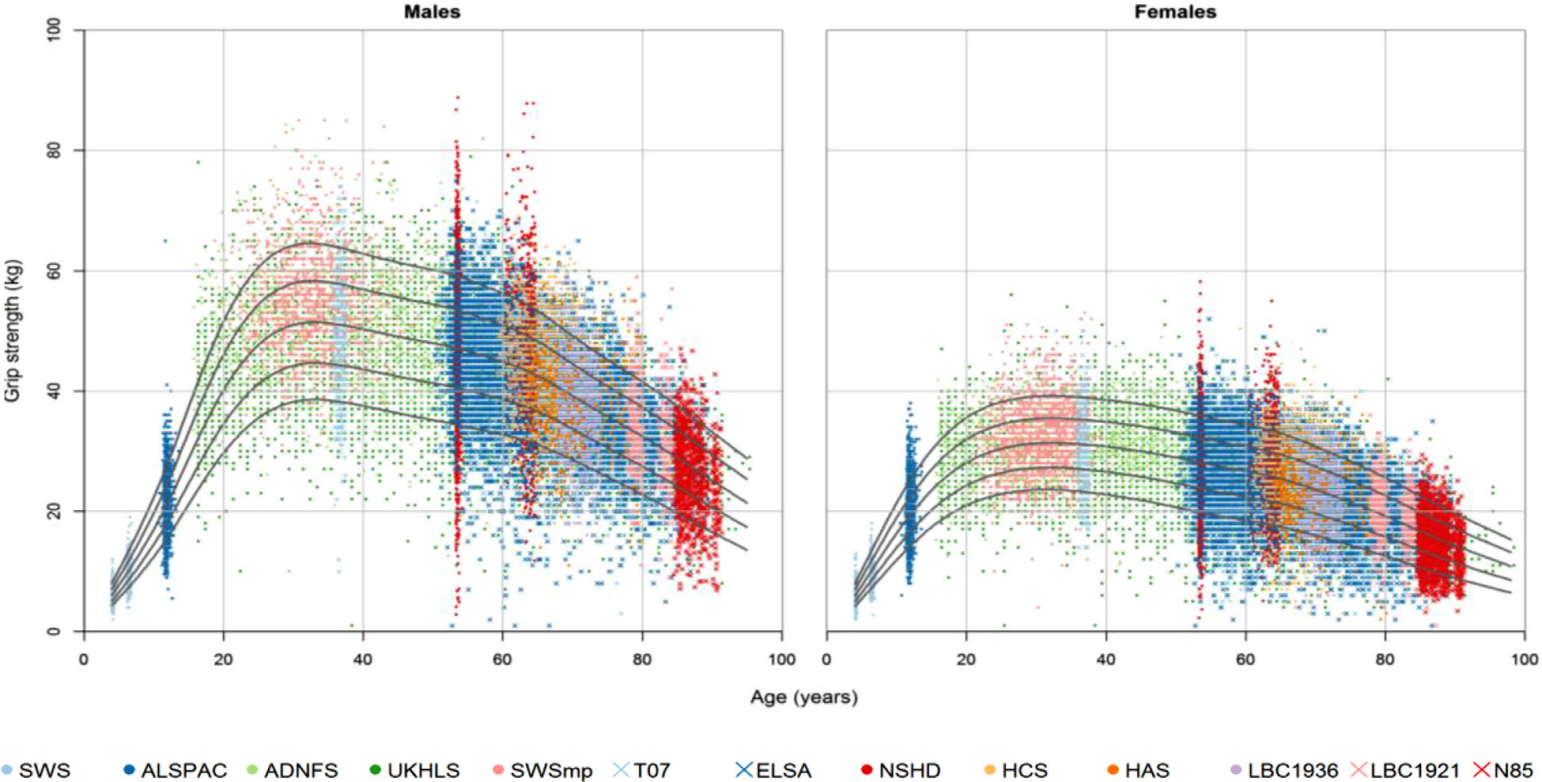
Grip strength across the life course in men and women. Used with permission from Dodds R, et al. Grip strength across the life course: normative data from 12 British birth and ageing cohorts. PLoS One. 2014;9(12):e113637.

This can be illustrated by a plot of an ageing biomarker such as grip strength against age in different individuals. There is a general rise to a peak in early adulthood followed by a plateau and then a decline in older age. However there is often considerable variation in the rate of decline and in part this reflects the effect of contemporaneous influences such as exercise, diet or ill health. However, later life muscle strength, and other ageing biomarkers, depends not only on the rate of loss but also on the peak reached in earlier life and in order to understand the peak, it is important to understand influences operating right from the earliest stages of life and throughout the subsequent years.^23^ This conceptual approach is borne out by normative data, albeit cross-sectional rather than longitudinal, published in a highly cited paper that for the first time brought together grip strength data from individuals in 12 British birth and ageing cohorts and plotted them against age (Fig. 5).^24^

Furthermore, exploiting the rich longitudinal data of UK birth cohorts, there is emerging evidence that interventions in mid-life can improve skeletal muscle in later life. The National Survey of Health and Development is a British birth cohort of over 5,000 singletons born in one week in March 1946 and followed up throughout life. Findings include evidence of cumulative benefits of increased physical activity across mid-life on grip strength at age 60–64 in men and women.^25^ This observational data suggest that mid-life physical activity may prevent decline in grip strength in early old age and could be used to inform intervention studies with the exciting possibility of integrating bench, bedside and lifecourse approaches for the prevention and treatment of ageing disorders.

## Future opportunities

The striking phrase ‘Medicine only advances with research’ opens chapter 9 of the 2023 chief medical officer report^6^ and there is opportunity for medicine to advance through translational ageing research. This needs growth not only in the number of geriatricians,^26^ but also in the number of academic geriatricians.^6^ Historically, the specialty has not had a strong academic base, but this is changing and there are now many centres of excellence around the country. Of course, addressing the complexity of ageing is going to need diversity of background and thought as well as discipline, and growing the workforce needs to go much wider than geriatric medicine.^27^

There is also the opportunity, through collaboration and investment, for UK-wide endeavour in this area. Making progress at scale and pace in translational ageing research is too big for any one centre and we already have many of the building blocks needed across the country to become a global leader. It is also an endeavour that goes beyond the academic community, and putting patients and the public at the centre of translational ageing research will be the key to maximising progress.^28^

Finally, there is the opportunity to apply translational ageing research to the needs of the NHS. Lord Darzi’s independent report on the NHS recognises the impact of an ageing population with rising multimorbidity.^29^ The government 10-year health plan proposes three big shifts in health and care – analogue to digital, hospitals to communities, and sickness to prevention.^30^ Translational ageing research has much to offer all three areas, but there may be a further opportunity. We know that integrating research into clinical services improves outcomes, so could we be bold and add a fourth big shift? I would like to propose that ageing research, and indeed all relevant research, be integrated as standard into practice throughout UK health and care. I think William Harvey would have approved.

## CRediT authorship contribution statement

**Avan Aihie Sayer:** Conceptualization, Writing – original draft, Writing – review & editing.

## Declaration of competing interest

The authors declare that they have no known competing financial interests or personal relationships that could have appeared to influence the work reported in this paper.
